# Spatial Distributions of HIV Infection in an Endemic Area of Western Kenya: Guiding Information for Localized HIV Control and Prevention

**DOI:** 10.1371/journal.pone.0148636

**Published:** 2016-02-10

**Authors:** Tomonori Hoshi, Yoshito Fuji, Samson Muuo Nzou, Chihiro Tanigawa, Ibrahim Kiche, Matilu Mwau, Anne Wanjiru Mwangi, Mohamed Karama, Kenji Hirayama, Kensuke Goto, Satoshi Kaneko

**Affiliations:** 1 Graduate School of Biomedical Sciences, Nagasaki University, Nagasaki, Japan; 2 Department of Ecoepidemiology, Institute of Tropical Medicine (NEKKEN), Nagasaki University, Nagasaki, Japan; 3 Nagasaki University Institute of Tropical Medicine–Kenya Medical Research Institute (NUTIM–KEMRI) Project, Nairobi, Kenya; 4 Centre for Infections and Parasitic Disease Control Research, Kenya Medical Research Institute (KEMRI), Busia, Kenya; 5 Thomas Odhiambo Campus, International Centre of Insect Physiology and Ecology (ICIPE), Mbita, Kenya; 6 Production Department, Kenya Medical Research Institute (KEMRI), Nairobi, Kenya; 7 Centre for Public Health Research, Kenya Medical Research Institute (KEMRI), Nairobi, Kenya; 8 Department of Immunogenetics, Institute of Tropical Medicine (NEKKEN), Nagasaki University, Nagasaki, Japan; 9 National Mental Support Center for School Children Crisis, Osaka Kyoiku University, Osaka, Japan; 10 Nagasaki University School of Tropical Medicine and Global Health, Nagasaki, Japan; University Hospitals of Leicester, UNITED KINGDOM

## Abstract

HIV is still a major health problem in developing countries. Even though high HIV-risk-taking behaviors have been reported in African fishing villages, local distribution patterns of HIV infection in the communities surrounding these villages have not been thoroughly analyzed. The objective of this study was to investigate the geographical distribution patterns of HIV infection in communities surrounding African fishing villages. In 2011, we applied age- and sex-stratified random sampling to collect 1,957 blood samples from 42,617 individuals registered in the Health and Demographic Surveillance System in Mbita, which is located on the shore of Lake Victoria in western Kenya. We used these samples to evaluate existing antibody detection assays for several infectious diseases, including HIV antibody titers. Based on the results of the assays, we evaluated the prevalence of HIV infection according to sex, age, and altitude of participating households. We also used Kulldorff’s spatial scan statistic to test for HIV clustering in the study area. The prevalence of HIV at our study site was 25.3%. Compared with the younger age group (15–19 years), adults aged 30–34 years were 6.71 times more likely to be HIV-positive, and the estimated HIV-positive population among women was 1.43 times larger than among men. Kulldorff’s spatial scan statistic detected one marginally significant (*P* = 0.055) HIV-positive and one significant HIV-negative cluster (*P* = 0.047) in the study area. These results suggest a homogeneous HIV distribution in the communities surrounding fishing villages. In addition to individual behavior, more complex and diverse factors related to the social and cultural environment can contribute to a homogeneous distribution pattern of HIV infection outside of African fishing villages. To reduce rates of transmission in HIV-endemic areas, HIV prevention and control programs optimized for the local environment need to be developed.

## Introduction

HIV is a major health problem in developing countries. Around two thirds of all HIV-infected individuals live in sub-Saharan Africa [1]. Several factors have been reported to contribute to the spread of HIV in this area, including the custom of polygamy, the non-use of condoms, cleansing rituals, and female genital mutilation [2–5]. In regions of Africa where fishing is the main industry, the transactional sexual practice referred to as “fish-for-sex” is recognized as one of the major risk behaviors for transmitting HIV infection [3, 6–8]. A substantial proportion of the population in African fishing communities are migrant workers who move from one village to another, and this behavior also promotes the spread of HIV infection.

To prevent the spread of HIV infection and improve the quality of life among people living with HIV, several approaches have been implemented, including condom provision, HIV/AIDS education programs, voluntary counseling and testing (VCT), harm reduction programs, and antiretroviral therapies [9]. As a result of these control and prevention efforts, the incidence of HIV infection has been declining in sub-Saharan Africa, especially among pregnant women [10]. However, despite the successful global reduction of HIV prevalence, vast discrepancies based on geographical area remain [11]. In Kenya, the estimated gap between the districts with the highest and lowest rates of HIV infection is 19.6% (21.0% versus 1.4%) [12]. In general, regions along Lake Victoria in western Kenya, where fishing is the primary industry, are associated with a higher prevalence of HIV infection [8, 13–17]. In such areas, “fish-for-sex” remains a common practice, and this might contribute to the transmission of HIV infection not only in the fishing communities, but also in the surrounding areas. However, even though knowledge of local HIV distribution patterns is important for developing effective prevention strategies, these patterns have not been well analyzed [18]. Therefore, in this study, we attempted to identify HIV hot/cold spots by using cluster analysis to observe distribution patterns of HIV infection in an area along Lake Victoria in western Kenya, which is known to have one of the highest HIV-endemic rates in the world [19].

## Methods

### Blood sampling

This study was conducted as part of data analyses in a population-based serological survey conducted at two Health and Demographic Surveillance System (HDSS) sites, the Mbita area site and the Kwale site; both sites are managed by the Institute of Tropical Medicine, Nagasaki University (NUITM), and the Kenya Medical Research Institute (KEMRI) [20]. The aim of this serological survey was to field test a simple and practical antibody detection assay system with a microsphere-based multiplex immunoassay system [19]. Among the total of 77,887 individuals (42,617 in Mbita, 35,270 in Kwale) registered in the HDSS, 4,600 individuals (2,300 individuals per site) were randomly selected according to HDSS site, sex, and age group. They were categorized by age as follows: those under the age of 45 years were grouped into five-year intervals, and those older than 45 years were consolidated as a single group. Next, 115 subjects were randomly selected from each sex and age group (0–4, 5–9,[…,] 45–75). In total, 2,300 participants across 20 sex-age strata were selected per site, and in this study we analyzed geo-referenced 1,957 samples from Mbita, where high levels of HIV prevalence were reported [19]. Blood was drawn from those who agreed to participate in the study at a blood sampling station, such as a school or health center, from July to August 2011.

### Microsphere-based multiplex immunoassays

All samples were sent to a laboratory in Nairobi and assayed to measure serum antibody levels against eight antigens derived from six pathogens, including three antigens derived from HIV (gag, gp120, and gp41). The details of our microsphere-based multiplex immunoassay system are described elsewhere [19]. Each of these antigens was coupled to a set of beads, and then exposed to each participant’s diluted serum to induce an immune response. To establish a cut-off point for each HIV antibody, we used sera from 40 HIV-negative Japanese individuals (reference). The serological scores were log_10_-transformed, and medians of the fluorescence intensities plus a 3-fold standard deviation against each antigen were used as cut-off values. Individuals who tested positive for three HIV-1 antigens were defined as positive. The details of this process are described elsewhere [19]. For spatial analysis, participant data from the Mbita site were extracted from the complete survey dataset because the Mbita site is a typical HIV-endemic area with many fishing villages.

**Fig 1:**
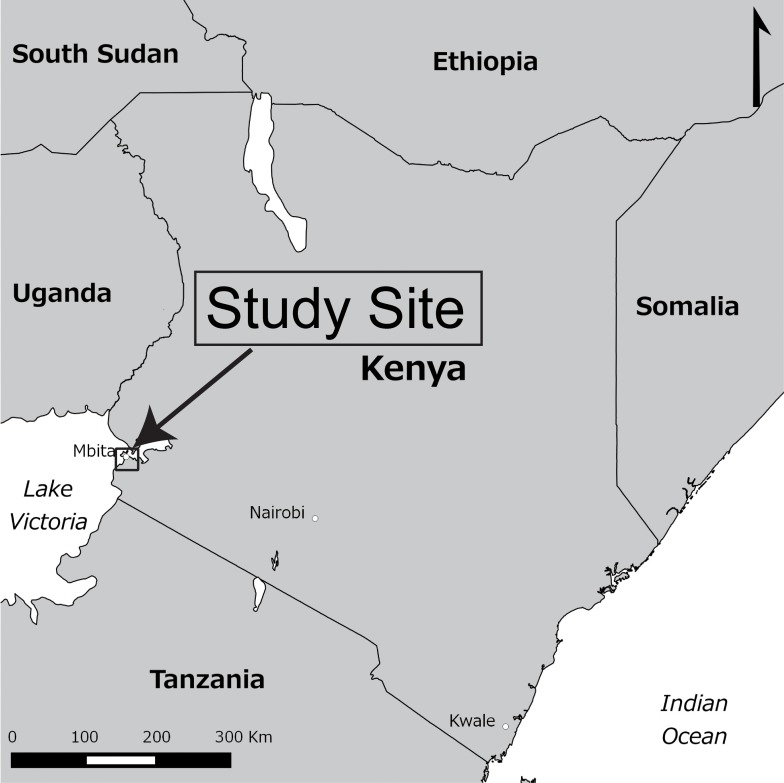
Study site along the shore of Lake Victoria in Mbita, Kenya. The HDSS program survey at the study site is managed by a NUITM-KEMRI Project.

The Mbita site is part of Homabay County in western Kenya, 310 km northwest of Nairobi, with an elevation of between 1,125 and 1,875 meters (Fig 1). The study site consisted of the following three sub-areas: (i) Rusinga Island (RI), a hilly geographical area with a small artificial land bridge to Gembe West; (ii) Gembe West (GW), an area that has the highest mountain among the sites; and (iii) Gembe East (GE), which has a relatively flat landscape and almost equally distributed households across the region.

All three areas face Lake Victoria. Approximately 50,000 residents from these three areas are registered under the HDSS program as reported previously [20]. The overall population of three sub-areas was 42,617 and population density was 260.1 people per square km (ppsk). RI was the densest area (396.2 ppsk) containing 16,611 people and followed by GE (234.7 ppsk, 11,072 people) and GW (165.9 ppsk, 12398 people). Population in RI distributed across the island, but the mountain in the middle of island had few inhabitants. In GE, the shore area had more population than the inland of the area. Population in GW homogeneously distributed across the area. Malaria is another major health problem in this area [21].

### Statistical analysis

To assess basic characteristics, HIV prevalence and estimated HIV-positive population were calculated according to sex and age in the three sub-areas using the proportions obtained from the survey and population data from the HDSS dataset.

To assess the distribution pattern and clusters of HIV hot/cold spots, we applied a generalized linear mixed model (GLMM) using binomial distribution (logistic generalized mixed model), with individual HIV status as the outcome. The best model for predicting factors affecting HIV prevalence was chosen after a backward stepwise model selection procedure was employed using Akaike’s information criterion (AIC). The full model included factors of age (ten strata), sex, altitude (100-meter increase), and region as fixed effects, i.e., explanatory variables, and households were considered a random effect, i.e., household-based analysis [22]. In addition, HIV prevalence and estimated HIV-positive population were computed using binomial confidence intervals. We also used Pearson's chi-squares to test the sampling equality in each sex-age stratum among the three regions. Analyses were done in R (version 2.15.3, 64 bit) via RStudio (version 0.97.320, 64 bit) using the glmmML package for GLMM and the binom package for binomial confidence intervals. A *P* value less than 0.05 was considered statistically significant.

To detect spatial clustering, we performed Kulldorff’s spatial scan statistic using SaTScan (version 9.2, 64 bit) [23]. This software has been widely applied in public health studies to identify the location of disease clusters [5, 24]. In brief, this software scans an entire study area with circular or elliptical windows and detects locations of disease clusters [25]. Here, clusters represent subpopulations that tend to have more/fewer reported cases, and we performed analyses for detecting low and high infection clusters, respectively. The maximum size of the scanning window can evaluate half of the study population. We set the maximum size of the scanning window to less than 50% of the total samples, which is a default setting for avoiding pre-selection bias. Both circular and elliptical window scans were performed to identify any differences between the results based on the types of scanning windows, and also we consider that detecting these two types of clusters in close proximity may be an indication that these are potential target areas where need interventions or resources are needed. We applied a Bernoulli model using the household coordinates (longitude and latitude) and HIV status (positive/negative) of each individual. The number of Monte Carlo replications was set as 9,999, but other settings or parameters were left at the default level. Spatial analysis was conducted at the household level as follows. A household that had more than one HIV-positive person was considered an HIV-positive household, since individuals in such households may be at a higher risk of HIV. If all members of a household were HIV negative, the household was categorized as an HIV-negative household. Using SaTScan, we located clusters of HIV-positive and HIV-negative households. The *P* value for SaTScan was set as less than or equal to 0.10. QGIS software (2.0.1, 64 bit) was used to map spatial data and display HIV distributions [26], and we the OpenStreetMap for the background image with the help of OpenStreetMap plug-in (available at: http://docs.qgis.org/1.8/en/docs/user_manual/osm/openstreetmap.html). Household altitudes were retrieved from the ASTER Global Digital Elevation Model (available at: http://gdem.ersdac.jspacesystems.or.jp) using the Raster interpolation plug-in (available at: http://3nids.github.io/rasterinterpolation). To map the distribution of HIV cases, 100-meter hexagon grids were created using the MMQGIS plug-in (available at: http://michaelminn.com/linux/mmqgis), and the ratios of HIV-positive households in each grid were shown.

### Ethical approval

In this study, we used data collected as part of a project involving the development of serological surveillance for tropical infectious diseases using simultaneous microsphere-based multiplex assays [19]. The protocol was approved by the Ethical Review Committee of the Kenya Medical Research Institute (KEMRI SSC No. 1934) and the Ethical Committee of the Institute of Tropical Medicine, Nagasaki University (10061550 and 10122261–2). We explained the project to all participants in advance, and obtained written informed consent before collecting blood samples. The consent for minors aged 0–12 years was obtained from their guardians or parents, and the assent was taken from adolescent aged 13–17. The consent and assent forms were either in English, Kiswahili, Luhya or Luo, and explanations were done with the language, which each participant could understand well.

## Results

**Fig 2:**
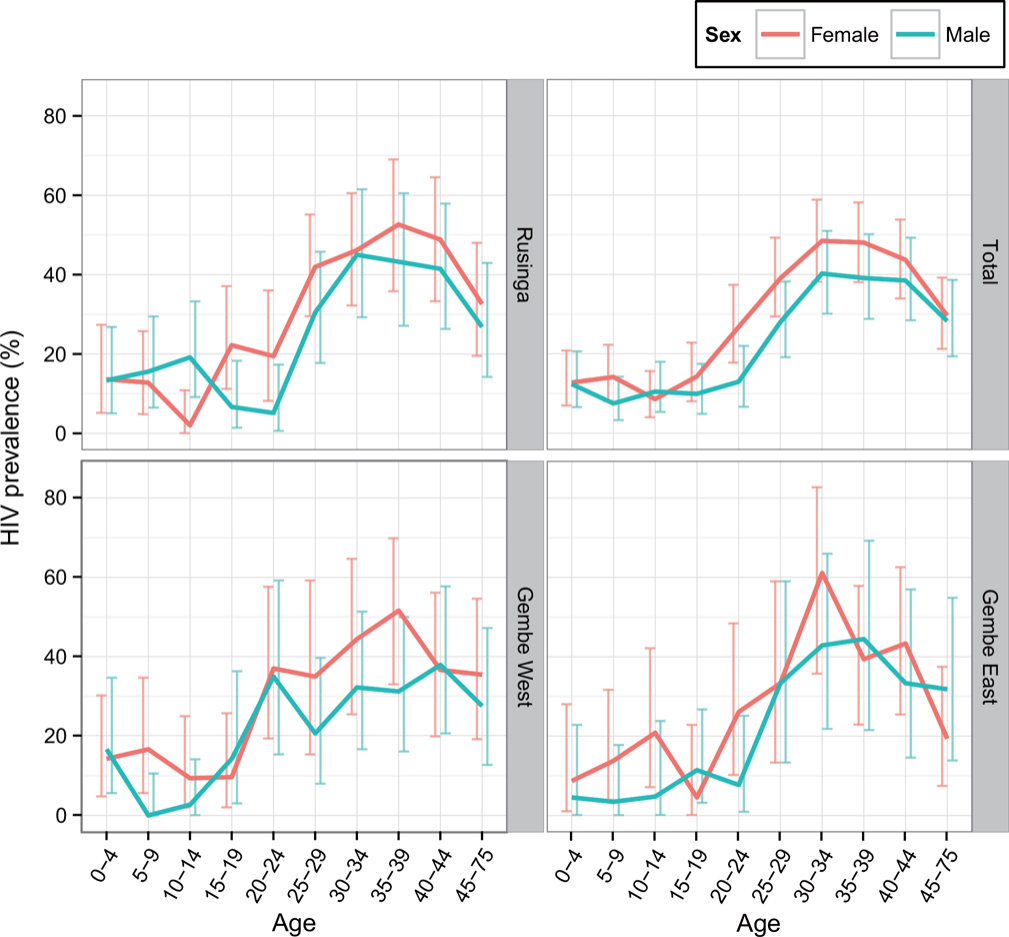
HIV prevalence by sub-area of the study site according to age-group and sex. The horizontal axis indicates age groups in units of five years, and the vertical axis shows the prevalence of HIV positive cases. Red and blue lines with error bars indicating 95% confidence intervals represent females and males, respectively.

A total of 1,957 geo-referenced blood samples were analyzed. An equal number of samples in each sub-area was collected from each sex and age group (chi-square test, Mbita (whole area): χ^2^(9) = 2.12, *P* = 0.989, RI: χ^2^(9) = 3.0458, *P* = 0.963, GE: χ^2^(9) = 10.475, *P* = 0.313, GW: χ^2^(9) = 5 87, *P* = 0.795). The mean HIV prevalence including all age- and sex- strata in Mbita was 25.34% (95% confidence interval [CI], 23.43–27.33), and all regional means were similar (Fig 2). The best GLMM model for factors affecting HIV infection included factors of sex, age, and altitude, but excluded region (Table 1). The odds ratio for HIV infection was 1.43 times higher among women than among men. Compared with the reference age group (15–19 years), the age group with the highest HIV prevalence was the 30–34 age group; this group was also 6.71 times more likely to be HIV positive. This group was followed in decreasing order of odds ratio by ages 35–39 (6.44-fold), ages 40–44 (5.75-fold), ages 25–29 (4.00-fold), and ages 45–75 (3.17-fold) (Table 2). Females between the ages of 30 and 34 had the highest HIV prevalence, especially in GE, where 61.11% (95% CI, 35.75–82.70) were HIV positive. Furthermore, boys between the ages of 5 and 9 years in GW were all HIV negative.

**Table 1:** Generalized linear mixed model (GLMM) for factors associated with HIV infection.

Factors	AIC	⊿AIC
Age, Sex, Altitude, Region	2027.81	3.75
**Age, Sex, Altitude**	**2024.06**	**0**
Age, Sex, Region	2031.11	7.05
Age, Altitude, Region	2035.03	10.97
Sex, Altitude, Region	2209.86	185.8
Age, Sex	2027.54	3.48
Age, Altitude	2031.29	7.23
Sex, Altitude	2206.9	182.84
Age	2034.59	10.53
Sex	2209.1	185.04
Altitude	2215.24	191.19
Region	2220.07	196.01

Each row shows the factors of each model. Enrolled individuals were grouped according to household.

AIC: Akaike’s information criterion

Δ: the difference with respect to the minimum value for AIC; **Minimum AIC is shown in bold.**

**Table 2:** Parameter estimates for a GLMM (binomial distribution) showing the influence of age, sex, and altitude on HIV positivity.

Factors	cOR^a^	Coef^b^	z score	P-value	AOR^c^	Coef^b^	z score	P-value
Age (years)								
0–4	1.06	0.05579	0.1760	0.86	1.03	0.027797	0.08725	0.93
5–9	0.89	-0.11473	-0.3572	0.721	0.886	-0.120789	-0.37436	0.708
10–14	0.76	-0.26854	-0.8111	0.417	0.772	-0.259076	-0.77918	0.436
15–19 (Reference)	1.00	-2.13013	2.0449	–	1.00	2.752924	1.22822	–
20–24	1.86	0.62271	-8.4433	0.0409	1.84	0.610447	1.99417	0.0461
25–29	4.01	1.38959	4.8505	<0.001	4.00	1.385876	4.80987	<0.001
30–34	6.59	1.88500	6.4447	<0.001	6.71	1.902968	6.45024	<0.001
35–39	6.42	1.86019	6.3836	<0.001	6.44	1.862783	6.34973	<0.001
40–44	5.75	1.74865	6.0246	<0.001	5.75	1.749860	5.98935	<0.001
45–75	3.14	1.14364	4.0278	<0.001	3.17	1.152619	4.03291	<0.001
Sex								
Male (Reference)	1.00	-1.35	-12.2	–	–	–	–	–
Female	1.43	0.356	3.16	0.0016	1.43	0.359	3.00	0.00273
Altitude								
Lowest altitude (Reference)	1.00	2.99	1.418	–	–	–	–	–
100 meter increase	0.699	-0.003583	-1.967	0.0491	0.645	-0.00438	-2.26	0.0239

^a^: crude odds ratio

^b^: coefficient

^c^: adjusted odds ratio.

Note: The best model for predicting HIV risk was chosen after a backward stepwise model selection procedure was employed using Akaike’s information criterion (AIC). The full model included factors of age (ten strata), sex, altitude (100-meter increase) and region as fixed effects, i.e., explanatory variables, and households were considered a random effect, i.e., household-based analysis.

**Fig 3:**
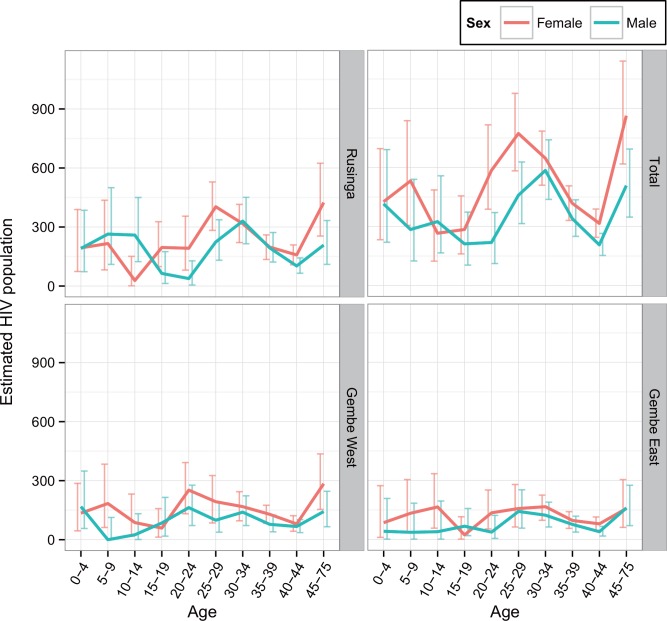
The estimated HIV positive population by sub-area of the study site according to age and sex. The horizontal axis shows five-year age groups, and the vertical axis shows the prevalence of HIV positive cases. Red and blue lines with error bars indicating 95% confidence intervals represent females and males, respectively.

Moreover, an approximate 35.5% decrease in HIV prevalence was evident with every 100-meter increase in altitude. The estimated HIV-affected population therefore comprised 8,666 (95% CI, 5,730–12,389) individuals, among which, females were about 1.44 times more likely than men (5,111 versus 3,555) to be HIV positive (Fig 3).

**Fig 4:**
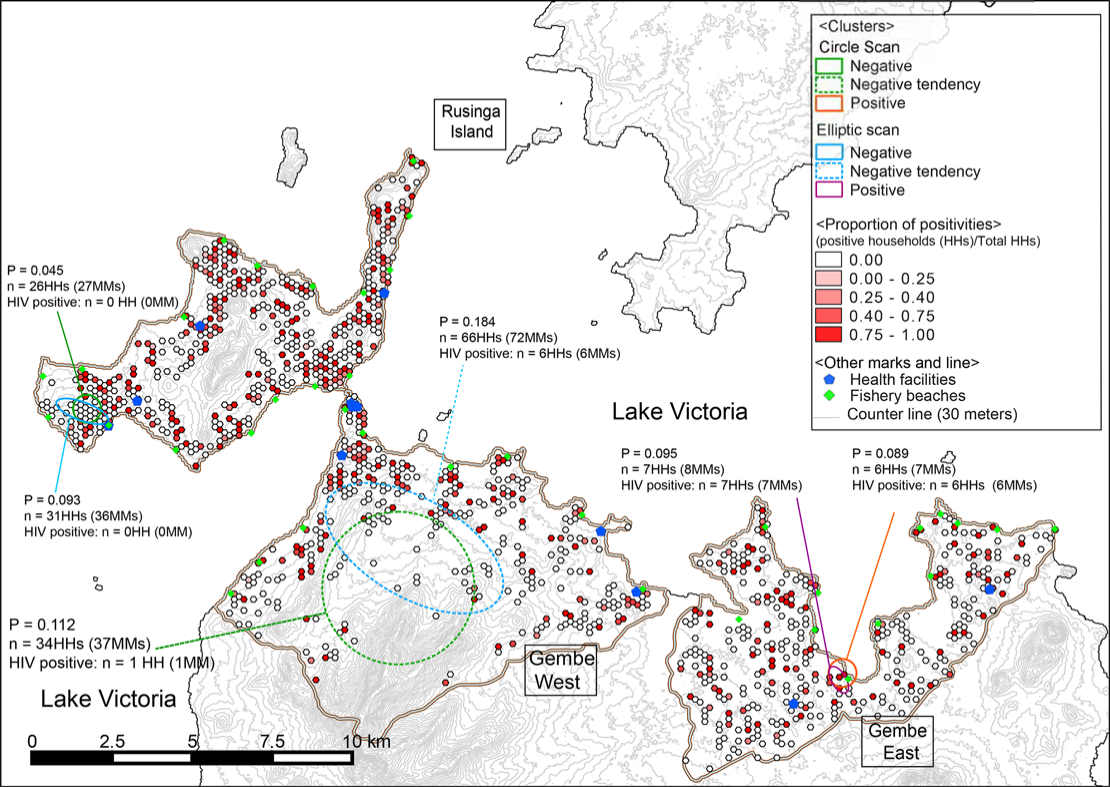
Results of cluster analysis using Kulldorff’s spatial scan statistic for both HIV-positive and negative clusters. Hexagons in color gradations from white to red represent proportions of HIV-positive households; blue pentagons represent health facilities; green diamonds represent fishing villages; circles with an orange solid line represent clusters of HIV positivity; circles with dotted lines represent clusters of HIV negative tendencies. Fig reused and superimposed the results of the cluster analyses on part of Fig from Fujii *et al*. [15]

The results of spatial clustering are shown in Fig 4. SaTScan circular scanning found one HIV-negative cluster in RI (*P* = 0.047), and one HIV-positive cluster in GE (*P* = 0.055). Elliptical scanning also found a negative cluster in RI (*P* = 0.093) and a positive cluster in GE (*P* = 0.095), and these were near the clusters found using circular scanning. The negative clusters were on the western side of the island and in a hilly area, whereas the positive clusters were located in a lowland area near Lake Victoria. Both negative and positive clusters had a total radius of about 430 meters; however, negative clusters contained more households (S1 Table: 26 versus 6 based using circular scanning; S2 Table: 31 versus 7, using elliptical scanning). In addition, one cluster with a slight HIV-negative tendency (not statistically significant) was identified in GW using circular scanning (Fig 4, *P* = 0.11), but no such tendency was identified using elliptical scanning (*P* = 0.18). The cluster with a negative tendency had 33 negative and one positive household in the circular window with a radius of 2,412.4 meters, which covered more than half of the area of the highest mountain in this region.

## Discussion

Based on the results of this study, we were able to identify detailed local patterns of HIV distribution in western Kenya. Few previous studies have been able to show HIV prevalence based on age, sex, geographical distribution, and clustering [5, 7]. The overall prevalence of HIV infection at our study site, 25.34%, was much higher than the nationwide average (5.6%) [27], but close to the estimated prevalence of the county (27%) [28]. Based on the population profile, these results suggest that there is a wide range of variation in HIV infection among sex- and age-based subpopulations (Fig 2). An extremely high prevalence of HIV infection was found among females aged 30–34 years in GE (61.11%). This high prevalence is nearly twice as much as the reported prevalence of 32.5% (95% CI, 25.8–39.3) among females of the same age in Asembo, approximately 30 km north-east of our study site [7], but close to the prevalence of widowed population 55.6 (95% CI, 51.0–60.0) in Ndhiwa, approximately 30km south-east of our study site [29]. Because GE area is located far from Mbita township; then this area deeply depends on traditional fishing economy compared with the other two areas in our study site. Therefore, females in this area might be more inclined to be involved in the transactional sex practice in fishery [3, 6–8], which may be related to this high HIV prevalence as widows who lost their husband by HIV in Ndhiwa. Furthermore, GE only had the HIV hot spot but without a cold spot, which might contribute to this higher HIV prevalence although we did not know an impact of having such hot spots in the same living area. While, women younger than 25 years of age tended to have a lower prevalence of HIV across sites, and age groups. The higher prevalence in the age group of 30–45 and the lower prevalence in the younger age groups suggests that individuals in sexually active age groups, especially females, have a higher risk of HIV infection than those in other regions and countries [30]. This pattern may be more pronounced due to the recent success in controlling HIV infection using antiretroviral treatments, which has led to improvements in survival and prevention of mother-to-child transmission [31], and thereby to changes in the age composition of HIV prevalence [32, 33].

To improve local prevention and control programs, the identification of geographical differences in HIV prevalence based on age and sex would provide vital information. For example, the prevalence of HIV in GW was found to be higher in the younger groups (age 20–24) of both sexes compared with other areas (RI and GE) in the same age group. This trend suggests locally existing risks of HIV transmission among younger populations specific to GW for both sexes. To improve the control program, situation analyses may be useful to identify the risks among the younger populations in this area compared with the other areas. Furthermore, the overall prevalence among children and females was high. The high prevalence among the 0–4 age group means that they are still infected vertically or during breastfeeding, and that earlier sexual initiation among girls than boys in this region would translates into higher HIV prevalence among young females compared with young males [34]. For the local optimization of the program, strategies aiming to prevent transmission to newborns during delivery or breastfeeding need to be strengthened [35]. In addition, we note an importance of prioritizing to use pre-exposure prophylaxis (PrEP) for the HIV negative population in the negative clusters, and also sexual active adolescents might be prioritized in such population [36].

Although a descriptive analysis of HIV trends in the study area is valuable from a public-health perspective, a spatial analysis adds value from the viewpoint of investigating HIV distribution patterns at the local level. We used SaTScan circular scanning to identify one HIV-positive cluster, one HIV-negative cluster, and one cluster with an HIV-negative tendency (Fig 4); the similar clusters, except for the negative tendency cluster, were identified using elliptical scanning. The statistically significant HIV-positive cluster was found along the shore of Lake Victoria in GE, where many fisheries are located and frequent transactional sex may occur [13]. This result was similar to those from previous studies that reported a high risk of HIV infection in fishing villages along Lake Victoria [15, 37]. No additional positive clusters were found in the other 37 fishing villages across the study area (Fig 4). This finding and the comparatively high prevalence in our study site suggests that the transmission of HIV infection extends beyond fishing villages and into the surrounding communities, with exception of the negative cluster. Unique cultural practices, including transactional sexual practices [13, 15], widow inheritance, cleansing rituals [38, 39], and internal female migration [14], may have contributed to the high prevalence of HIV seen in the fishing communities near Lake Victoria. Furthermore, the recent and rapid proliferation of motorbike taxis in the study area may also have contributed to the spread of HIV to the surrounding areas. This is because motorbike taxi drivers tend to engage in sexual practices similar to those in fishing communities (personal communication with a researcher) [40], which could accelerate disease transmission beyond the fishing communities. The same risk behaviors have been reported among taxi drivers in Ethiopia [41].

Partnership interventions to break transmission chains or reduce the risk of transmission from infected persons (often called the index case) to their uninfected partners have become a major strategy to reduce the prevalence of sexually transmitted infections globally [42]. In Kenya, a new program that promotes partnership interventions has recently started [43]. Although the results from this intervention will in part function to encourage HIV testing and treatment, considering the complex environments of communities in HIV-endemic areas, multimodal programs optimized culturally, environmentally, and occupationally to the local situation need to be developed to prevent HIV transmission in the communities where HIV transmission networks are complex and diverse [44]. HIV transmission between household members may not be clearly evaluated in our study site. We classified a household with more than one infected individual as a positive household in this study, which may potentially have led to an overestimation of HIV infection.

Two negative clusters appeared in hilly areas, which was consistent with the best GLMM model that suggested a decrease of approximately 35.5% in HIV prevalence for every 100-meter increase in altitude (Table 2); the other negative cluster was not in a hilly area. The negative clusters in hilly areas could be explained by the fact that these areas are isolated and the residents have less social interaction compared with other areas where high risk behaviors are practiced [16, 45], even though the other negative cluster was not located in a hilly area or surrounded by areas showing a homogeneous distribution of HIV infection. This negative cluster may have other undefined preventive factors which could represent a potential solution for the surrounding areas with a high prevalence of HIV because these areas all share cultural and environmental factors. Further studies are necessary to identify the undefined preventive factors in this area.

In summary, the HIV distribution pattern identified in the study area was shown to be homogeneous beyond the fishing villages. This is thought to be attributable to the complex and diverse cultural environments in the study area, as well as changing economic patterns. To develop optimal strategies for the prevention and control of HIV transmission in such communities, social and environmental factors in addition to those related to fishing should be considered.

## Supporting Information

S1 Table Results of cluster analysis by circle scan mode. (DOCX)

S2 Table Results of cluster analysis by Elliptic Medium scan. (DOCX)
