# Supplementary material for: Spatial Distributions of HIV Infection in an Endemic Area of Western Kenya: Guiding Information for Localized HIV Control and Prevention
Source: PLoS One. 2016 Feb 10;11(2):e0148636. doi: 10.1371/journal.pone.0148636 (PMC4749294; doi:10.1371/journal.pone.0148636)
Supplement: S1 Table — (DOCX) [file pone.0148636.s001.docx]

Table 1S. Circle scan mode

| Area | Scan type | Order | Radius (meter) | Population (case) | Expected case | Observed / expected case | Relative risk | P-value |
| --- | --- | --- | --- | --- | --- | --- | --- | --- |
| Rusinga | High | 1 | 485.78 | 14 (11) | 4.13 | 2.67 | 2.75 | 0.333 |
|  |  | 2 | 366.73 | 5 (5) | 1.47 | 3.39 | 3.45 | 0.733 |
|  |  | 3 | 385.16 | 5 (5) | 1.47 | 3.39 | 3.45 | 0.733 |
|  | Low | 1 | 433.25 | 26 (0) | 7.66 | 0 | 0 | 0.045** |
| Gembe West | High | 1 | 728.59 | 21 (13) | 5.60 | 2.32 | 2.46 | 0.560 |
|  |  | 2 | 254.62 | 12 (8) | 3.2 | 2.5 | 2.59 | 0.940 |
|  | Low | 1 | 2412.41 | 34 (1) | 9.07 | 0.11 | 0.10 | 0.112 |
|  |  | 2 | 994.14 | 16 (0) | 4.27 | 0 | 0 | 0.698 |
|  |  | 3 | 610.00 | 15 (0) | 4.00 | 0 | 0 | 0.810 |
|  |  | 4 | 161.34 | 14 (0) | 3.73 | 0 | 0 | 0.917 |
|  |  | 5 | 1026.18 | 13 (0) | 3.47 | 0 | 0 | 0.982 |
|  |  | 6 | 454.45 | 12 (0) | 3.20 | 0 | 0 | 0.998 |
| Gembe East | High | 1 | 435.66 | 6 (6) | 1.52 | 3.95 | 4.13 | 0.089* |
|  |  | 2 | 412.99 | 4 (4) | 1.01 | 3.95 | 4.07 | 0.766 |
|  |  | 3 | 389.43 | 8 (6) | 2.02 | 2.97 | 3.08 | 0.912 |
|  |  | 4 | 532.16 | 3 (3) | 0.76 | 3.95 | 4.04 | 0.996 |
|  |  | 5 | 495.77 | 3 (3) | 0.76 | 3.95 | 4.04 | 0.996 |
|  | Low | 1 | 869.57 | 20 (0) | 5.06 | 0 | 0 | 0.337 |
|  |  | 2 | 744.57 | 16 (0) | 4.05 | 0 | 0 | 0.787 |
|  |  | 3 | 684.87 | 14 (0) | 3.54 | 0 | 0 | 0.938 |
|  |  | 4 | 1670.02 | 13 (0) | 3.29 | 0 | 0 | 0.975 |
|  |  | 5 | 699.56 | 10 (0) | 2.53 | 0 | 0 | 0.999 |
|  |  | 6 | 752.25 | 10 (0) | 2.53 | 0 | 0 | 0.999 |
|  |  | 7 | 693.50 | 10 (0) | 2.53 | 0 | 0 | 0.999 |
