# Supplementary material for: Spatial Distributions of HIV Infection in an Endemic Area of Western Kenya: Guiding Information for Localized HIV Control and Prevention
Source: PLoS One. 2016 Feb 10;11(2):e0148636. doi: 10.1371/journal.pone.0148636 (PMC4749294; doi:10.1371/journal.pone.0148636)
Supplement: S2 Table — (DOCX) [file pone.0148636.s002.docx]

Table 2S. Elliptic Medium scan

| Area | Scan type | Order | Semi-minor axis (meter) | Semi-major axis (meter) | Angle | Population (case) | Expected case | Relative risk | P-value |
| --- | --- | --- | --- | --- | --- | --- | --- | --- | --- |
| Rusinga | High | 1 | 350.67 | 701.34 | 0 | 24 (17) | 7.07 | 2.52 | 0.295 |
|  |  | 2 | 191.75 | 767.0 | -45 | 8 (8) | 2.36 | 3.48 | 0.469 |
|  |  | 3 | 482.10 | 1446.30 | 80 | 12 (10) | 3.54 | 2.91 | 0.837 |
|  |  | 4 | 136.48 | 545.93 | -30 | 5 (5) | 1.47 | 3.45 | 0.999 |
|  |  | 5 | 93.83 | 140.75 | 90 | 4 (4) | 1.18 | 3.44 | 0.999 |
|  | Low | 1 | 285.56 | 856.69 | -20 | 31 (0) | 9.14 | 0 | 0.093* |
|  |  | 2 | 468.52 | 1405.55 | 60 | 16 (0) | 4.72 | 0 | 0.999 |
|  |  | 3 | 217.48 | 434.96 | 60 | 14 (0) | 4.13 | 0 | 0.999 |
| Gembe West | High | 1 | 728.59 | 728.59 | 0 | 12 (13) | 5.60 | 2.46 | 0.864 |
|  |  | 2 | 23.27 | 46.53 | 60 | 4 (4) | 1.07 | 3.83 | 0.997 |
|  |  | 3 | 39.26 | 117.78 | 40 | 4 (4) | 1.07 | 3.83 | 0.999 |
|  |  | 4 | 254.62 | 254.62 | 0 | 12 (8) | 3.20 | 2.59 | 0.999 |
|  | Low | 1 | 1530.92 | 3061.84 | -30 | 66 (5) | 17.60 | 0.26 | 0.184 |
|  |  | 2 | 437.92 | 1751.70 | 60 | 21 (0) | 5.60 | 0 | 0.931 |
|  |  | 3 | 766.49 | 1149.73 | 90 | 17 (0) | 4.53 | 0 | 0.942 |
|  |  | 4 | 161.34 | 161.34 | 0 | 14 (0) | 3.73 | 0 | 0.998 |
|  |  | 5 | 521.12 | 2084.50 | 30 | 17 (0) | 4.53 | 0 | 0.999 |
| Gembe East | High | 1 | 228.81 | 457.62 | -60 | 7 (7) | 1.77 | 4.16 | 0.095* |
|  |  | 2 | 400.27 | 600.40 | 90 | 6 (6) | 1.52 | 4.13 | 0.254 |
|  |  | 3 | 601.28 | 1803.83 | -80 | 5 (5) | 1.26 | 4.10 | 0.850 |
|  |  | 4 | 408.44 | 612.66 | 0 | 9 (7) | 2.28 | 3.22 | 0.947 |
|  |  | 5 | 265.87 | 531.74 | 0 | 4 (4) | 1.01 | 4.07 | 0.985 |
|  |  | 6 | 495.77 | 495.77 | 0 | 3 (3) | 0.76 | 4.04 | 0.999 |
|  | Low | 1 | 869.57 | 869.57 | 0 | 20 (0) | 5.06 | 0 | 0.728 |
|  |  | 2 | 744.57 | 744.57 | 0 | 16 (0) | 4.05 | 0 | 0.987 |
|  |  | 3 | 441.83 | 1767.30 | 75 | 19 (0) | 4.81 | 0 | 0.994 |
|  |  | 4 | 1564.15 | 3128.30 | -30 | 16 (0) | 4.05 | 0 | 0.996 |
|  |  | 5 | 684.87 | 684.87 | 0 | 14 (0) | 3.54 | 0 | 0.999 |
|  |  | 6 | 554.41 | 2772.03 | 12 | 18 (0) | 4.55 | 0 | 0.999 |
